# Evaluating the Efficacy of Combining Sensory Room and Conventional Therapies for Lebanese Children With Autism: A 10-Year Study

**DOI:** 10.7759/cureus.69953

**Published:** 2024-09-22

**Authors:** Issa Awaida, Aalaa A Saleh, Jad El Masri, Solay Farhat, Oulfat El Tourjouman

**Affiliations:** 1 Department of Pediatrics, Lebanese University, Beirut, LBN; 2 Department of General Medicine, Lebanese University, Beirut, LBN

**Keywords:** autism spectrum disorder (asd), conventional therapy, parental concerns questionnaire inferring alterations (pcqia), sensory integration, sensory room therapy

## Abstract

Background

Autism spectrum disorder (ASD) is a developmental condition characterized by sensory difficulties, which pose a significant challenge. Our aim is to evaluate the effectiveness of sensory room therapy in conjunction with traditional therapy, comparing it to traditional therapy alone, among children diagnosed with ASD in Lebanon.

Methods

A retrospective longitudinal study with cross-sectional survey (mixed study) was conducted over a 10-year period, involving 548 children diagnosed with autism spectrum disorder (ASD). The children were divided into two groups: group 1, comprising 306 children who received conventional therapy in addition to sensory room therapy, and group 2, consisting of 242 children who received conventional therapy alone. Data collection included sociodemographic characteristics, autism-related features, and scores from the Parental Concerns Questionnaire Inferring Alterations (PCQIA) scale, which measures sensory and behavioral characteristics. Data were collected at two time points: before the initiation of sensory room therapy and after the completion of the therapy, allowing for an assessment of changes and effectiveness post-treatment.

Results

In both groups, there was no significant difference in PCQIA scores following conventional therapy alone (group 1: mean score 34.1, group 2: mean score 33.4; p=0.222). However, a significant increase in PCQIA scores was observed after the addition of sensory room therapy in group 1 (combined therapy), with scores rising from a pre-therapy mean of 34.1 to a post-therapy mean of 41.7 (p<0.001). Moreover, there was a positive correlation between PCQIA scores and parental income. Additionally, 78.2% of parents rated sensory room therapy as highly effective, with 62% reporting significant improvement in their children’s behavior and 80% noting increased engagement in extracurricular activities. Notably, 98% of parents indicated they would recommend the combined therapy to others.

Conclusion

Sensory room therapy demonstrates improvement in sensory challenges and motor skills among children diagnosed with ASD.

## Introduction

Autism spectrum disorder (ASD) is a complex neurodevelopmental condition characterized by deficits in social communication, repetitive behaviors, restricted interests, and strict adherence to routines [1]. Despite the inherent challenges in its clinical delineation, studies suggest that between 42% and 88% of individuals with ASD experience sensory processing impairments, exhibiting both hyper- and hypo-responsiveness [2,3]. In Lebanon, a middle-income country in the Middle East, national prevalence studies on ASD are limited. However, a recent study conducted in the Beirut and Mount Lebanon governorates using the Modified Checklist for Autism in Toddlers (M-CHAT) estimated the prevalence of ASD at 153 per 10,000 children aged 16-48 months in nurseries [4].

Beyond core symptoms, sensory processing difficulties impose a significant burden on children with ASD and their families. These challenges often manifest as difficulties in managing sensory stimuli such as noise, touch, movement, taste, and sight, contributing to behavioral problems and daily functioning limitations [5]. Consequently, addressing sensory processing issues has become an integral component of conventional therapeutic approaches, including applied behavioral analysis (ABA), speech therapy, psychomotor therapy, occupational therapy, and psychotherapy [6].

Occupational therapy, in particular, has evolved to better accommodate the sensory and environmental needs of children with ASD, resulting in the establishment of sensory-specific interventions [7]. Multisensory rooms (also known as sensory rooms), first introduced in the 1960s, were initially designed to provide individuals with ASD with demand-free sensory stimulation, enriching sensory input and promoting relaxation and self-regulation [8]. While the original concept focused on delivering basic sensory stimuli - targeting sight, smell, sound, and touch - recent advancements have significantly expanded the application of sensory rooms. These modern interventions now incorporate interactive projections, adaptive lighting, and advanced tactile equipment, creating personalized therapeutic environments [9].

Although the sensory room concept has existed for decades, its application in modern therapeutic practices has evolved. There is growing global interest in integrating sensory therapy with conventional treatments such as ABA and occupational therapy. In fact, sensory rooms have been shown to promote skill development in communication, motor coordination, and emotional regulation, making them a valuable adjunct to conventional therapy [10]. Despite these promising results, long-term studies on the efficacy of sensory rooms remain scarce, especially in low to middle income countries including Lebanon, where data on sensory interventions for autism is limited. This gap underscores the need for research in regions where socioeconomic factors may influence the availability and quality of therapeutic interventions. 

This study aims to evaluate the effectiveness of combining sensory room therapy with conventional therapy in improving sensory processing, behavioral outcomes, and skill development in Lebanese children with ASD, as measured by changes in Parental Concerns Questionnaire Inferring Alterations (PCQIA) scores and parental satisfaction levels. Additionally, the study seeks to compare the changes in PCQIA scores and parental satisfaction between children receiving combined therapy versus conventional therapy alone. It also aims to assess the effectiveness and safety of sensory room interventions in addressing sensory and behavioral challenges in ASD, while analyzing correlations between family income levels and treatment outcomes. The findings from this study are expected to provide data that may inform future clinical practices and public health strategies for managing ASD in Lebanon and in similar low- to middle-income countries across the world. 

## Materials and methods

Setting, study design, and participants

This study employed a retrospective longitudinal design combined with a cross-sectional survey approach (a mixed-methods study) conducted over a 10-year period, from 2010 to 2020, involving 548 children diagnosed with autism spectrum disorder (ASD). The children included in the study were registered at five health and educational centers across Lebanon. Inclusion criteria were applied to select Lebanese autistic children who were receiving therapy before 2020 and were aged between four and 12 years at the time of therapy initiation. Patients who began therapy after the age of 12 were excluded from the study.

To investigate the impact of sensory room therapy in conjunction with conventional therapy, the participants were categorized into two groups based on their treatment history: group 1 (N=306) consisted of children who received conventional therapy in addition to sensory room therapy, and group 2 (N=242) consisted of children who received conventional therapy alone.

Data source and measures

Data were collected retrospectively through health and therapy records, alongside a cross-sectional survey administered to the parents at the end of the study period. The survey included the Parental Concerns Questionnaire Inferring Alterations (PCQIA) scale, which measures sensory and behavioral characteristics. The data collection was done at two points: before the initiation of sensory room therapy (for group 1) and at the end of the study period for both groups, assessing changes in sensory processing, behavior, and overall effectiveness of the combined therapy. A fully-comprehensive data collection form was designed and implemented using Google Forms (Google, Mountain View, California). Data were collected through detailed interviews with parents or caregivers of included children with ASD. The collected data encompassed various variables, including socio-demographic characteristics like gender, age, and monthly income; autism-related characteristics such as diagnosis, communication and language abilities, compulsive behaviors/restrictive interests, and social interaction; the Parenteral Concerns Questionnaire Inferring Alteration Scale (PCQIA), which is a scale inspired by the Parenteral Concerns Questionnaire (PCQ) [11] used to assess the efficacy of treatment. This section consisted of 13 items assessing variations in symptoms related to communication, behavior, anxiety, sensory issues, sleep, aggression, hyperactivity, attention, mood, eating habits, social interaction, self-stimulation, and self-injury. PCQIA was utilized in both study groups; and the efficacy of sensory room, which included questions directed at parents whose children attended sensory and conventional therapy sessions, addressing the efficacy of sensory room interventions, potential drawbacks, changes in symptoms, improvements, engagement in extracurricular activities, and recommendations for other children with ASD.

Ethical considerations

Ethical approval was obtained from the Institutional Review Board (IRB) at Rafik Hariri University Hospital in Beirut, Lebanon. Informed consent was obtained from the managers of each center/school as well as from the patient's parents/legal guardians, and participation was voluntary for all patients. Confidentiality of participant information was assured.

Statistical analysis

All statistical analyses were conducted using SPSS version 25.0 (IBM Inc., Armonk, New York). Descriptive statistics included mean, standard deviation (SD), median, interquartile range, minimum and maximum values, and 95% confidence limits (CLs) for continuous data. Categorical variables were presented as absolute and relative frequencies (n and %). Nonparametric tests such as the Mann-Whitney test, K-sample independent test, Kruskal-Wallis test, and Wilcoxon Signed Ranks Test were employed for bivariate analysis. A significance level of p<0.05 was used to determine statistical significance.

The newly developed PCQIA scale demonstrated strong internal consistency with a Cronbach's Alpha of 0.9, adequate sampling adequacy with a Kaiser-Meyer-Olkin (KMO) measure of 0.881, and significant sphericity according to Bartlett's Test (p<0.001).

## Results

Sociodemographic characteristics

The distribution of study groups based on gender (p=0.842) and age (p=0.474) was nearly equal. In group 1, there were 210 males (68.63%) and 96 females (31.37%), a distribution similar to that of group 2, which comprised 168 males (69.42%) and 74 females (30.58%; Table 1).

**Table 1:** Representation of the study groups in function of their sociodemographic characteristics Group 1 - sensory + traditional; Group 2 - traditional

Sociodemographic characteristics		Therapy group		Total (N=548)	P-value
		Group 1 (N=306)	Group 2 (N=242)		
Gender	Male	210	168	378	0.842^ a^
		68.63%	69.42%	68.98%	
	Female	96	74	170	
		31.37%	30.58%	31.02%	
Age	Mean (SD)	8.29 (1.96)	8.44 (2.83)	8.36 (2.39)	0.474^ b^
	Min - Max	13-Apr	20-Apr	20-Apr	
Age when the patient joined the center	Mean (SD)	5.11 (1.90)	5.07 (1.11)	5.09 (1.60)	0.785^ b^
	Min - Max	10-Feb	8-Mar	10-Feb	

Communication and language

Among the 548 children included in the study, 82% exhibited poor spoken language, 2% demonstrated poor augmentative communication, 66.42% struggled with engaging in conversations with others, and 24.45% displayed limited use of gestures. There was no significant difference between the two groups regarding these factors (Table 2).

**Table 2:** Representation of the study groups in function of the communication and language use Group 1 - sensory + traditional; Group 2 - traditional

Communication and language use		Therapy group		Total (N=548)	P-value
						Group 1 (N=306)	Group 2 (N=242)
		Poor spoken language	No			56	38	94	0.423
18.30%	15.70%			17.15%					
Yes	250		204	454					
	81.70%		84.30%	82.85%					
Poor augmentative communication	No	300	238	538	1.000
		98.04%	98.35%	98.18%	
	Yes	6	4	10	
		1.96%	1.65%	1.82%	
Poor conversations with others	No	102	82	184	0.892
		33.33%	33.88%	33.58%	
	Yes	204	160	364	
		66.67%	66.12%	66.42%	
Low gestures	No	240	174	414	0.077
		78.43%	71.90%	75.55%	
	Yes	66	68	134	
		21.57%	28.10%	24.45%	

Compulsive behaviors/restrictive interests

Both groups of children exhibited inflexible adherence to routines (p=0.280), repetitive body movements (p=0.015), compulsions and rituals (p=0.022), and unusual preoccupations (p=0.082; Table 3).

**Table 3:** Representation of the study groups in function of the compulsive behaviors Group 1 - sensory + traditional; Group 2 - traditional

Compulsive behaviors		Therapy group		Total (N=548)	P-value
						Group 1 (N=306)	Group 2 (N=242)
		Inflexible adherence to routines	No			168	144	312	0.280
54.90%	59.50%			56.93%					
Yes	138		98	236					
	45.10%		40.50%	43.07%					
Repetitive body movements	No	156	98	254	0.015
		50.98%	40.50%	46.35%	
	Yes	150	144	294	
		49.02%	59.50%	53.65%	
Compulsions and rituals	No	240	214	454	0.002
		78.43%	88.43%	82.85%	
	Yes	66	28	94	
		21.57%	11.57%	17.15%	
Unusual preoccupations	No	240	204	444	0.082
		78.43%	84.30%	81.02%	
	Yes	66	38	104	
		21.57%	15.70%	18.98%	

Social interactions

There were no significant differences between the two groups in terms of poor eye contact (p=0.111), lack of peer relationships (p=0.031), absence of shared interests with others (p=0.315), and non-participation in social play or games (p=0.506; Table 4).

**Table 4:** Representation of the study groups in function of social interaction Group 1 - sensory + traditional; Group 2 - traditional

Social interaction		Therapy group		Total (N=548)	P-value
						Group 1 (N=306)	Group 2 (N=242)
		Poor eye contact	No			124	82	206	0.111
40.52%	33.88%			37.59%					
Yes	182		160	342					
	59.48%		66.12%	62.41%					
Peer relationships (prefer to be alone)	No	208	208	416	0.03
		67.97%	85.95%	75.91%	
	Yes	98	34	132	
		32.03%	14.05%	24.09%	
Lack of sharing interests with others	No	180	132	312	0.315
		58.82%	54.55%	56.93%	
	Yes	126	110	236	
		41.18%	45.45%	43.07%	
Not participate in social play or games	No	304	242	546	0.506
		99.35%	100.00%	99.64%	
	Yes	2	0	2	
		0.65%	0.00%	0.36%	
No problems	No	292	232	524	0.801
		95.42%	95.87%	95.62%	
	Yes	14	10	24	
		4.58%	4.13%	4.38%	

Parental Concerns Questionnaire Inferring Alteration (PCQIA)

**Figure 1:**
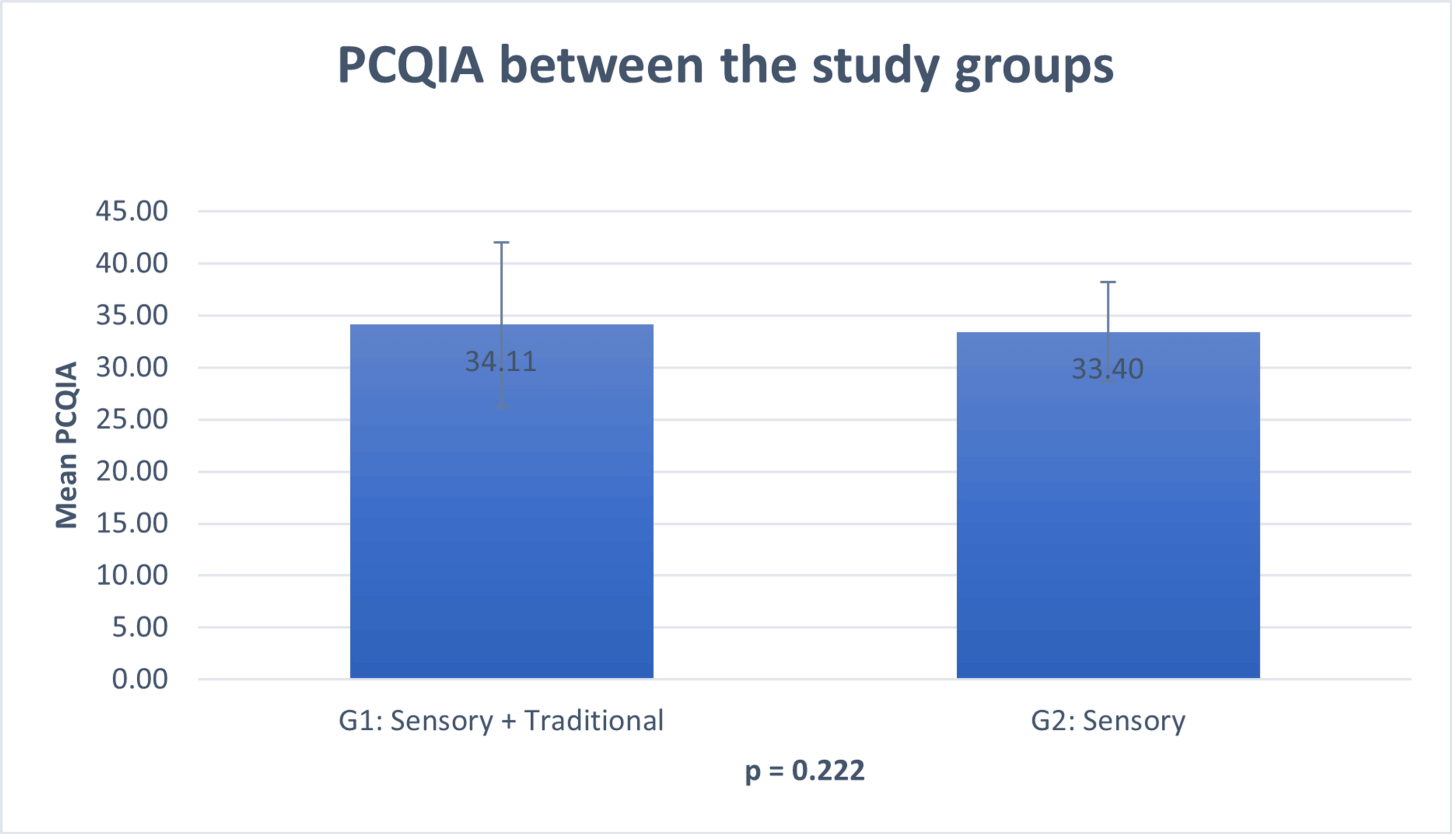
Mean PCQIA in function of the two study groups after the conventional therapy PCQIA - Parental Concerns Questionnaire Inferring Alterations

**Figure 2:**
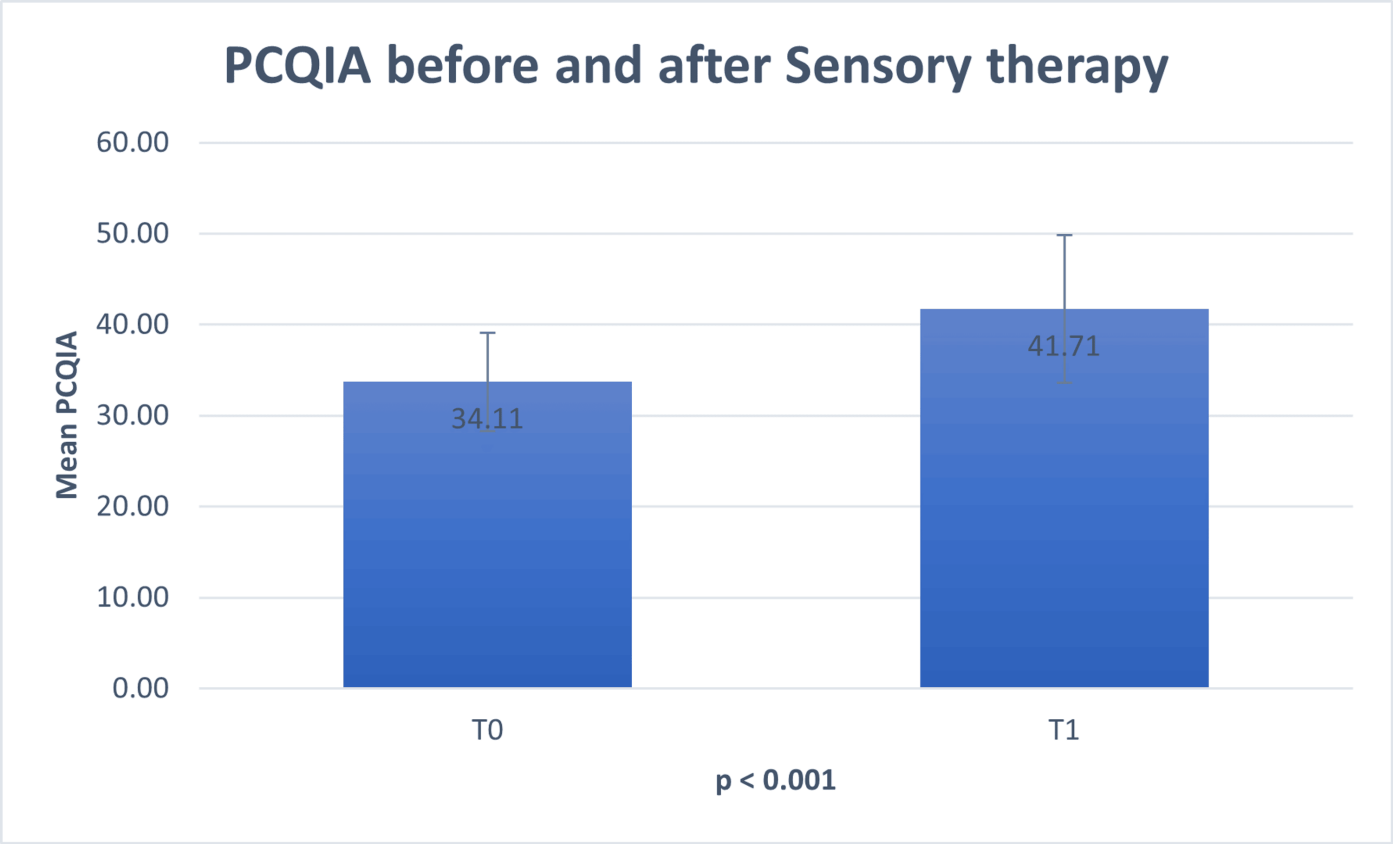
Mean difference of PCQIA scale before and after therapy in children who underwent mixed therapy PCQIA - Parental Concerns Questionnaire Inferring Alterations

Following conventional therapy, there was no statistically significant difference in PCQIA scores between the two study groups (p=0.222). The mean PCQIA score was 34.1 in group 1 and 33.40 in group 2 (Figure 1). The Wilcoxon Signed Ranks Test revealed a significant increase in PCQIA scores post-therapy for children who underwent both sensory and traditional therapy (p<0.001). The mean score before therapy was 34.11 out of 52, which increased to 41.7 out of 52 (Figure 2).

Illnesses

There was no significant difference between the two groups in terms of illness (p=0.017). Among the 548 children in the study, 43.1% suffered from a medical illness associated with autism disorder. The digestive system was the most commonly affected system, with 125 cases (Table 5).

**Table 5:** Representation of the study groups in function of illness Group 1 - sensory + traditional; Group 2 - traditional

Associated comorbidities		Therapy group		Total (N=548)	P-value
		Group 1 (N=306)	Group 2 (N=242)		
Does the patient suffer from any medical illness associated to autism disorder?	No	188	124	312	0.017 ^a^
		61.4%	51.2%	56.9%	
	Yes	118	118	236	
		38.6%	48.8%	43.1%	
Type of medical illness associated to autism disorder	Respiratory system	14	24	38	
	Digestive system	70	104	174	
	Metabolic system/ mitochondrial diseases/ others	6	0	6	
	Urinary and reproductive system	12	12	24	
	Lymphatic system	22	0	22	
	Allergic and/or immunological system	10	58	68	
	Genetic disorder (Rett syndrome / fragile X syndrome / other)	6	0	6	
	Motor deficit: cerebral palsy/ hemiplegia/ hypotonia/spasticity/ others	8	0	8	
	Sensory deficit (visual problem /auditory problem/ others)	46	70	116	
	Seizure	10	0	10	
	Cerebral palsy	6	0	6	

Efficiency of sensory room

**Figure 3:**
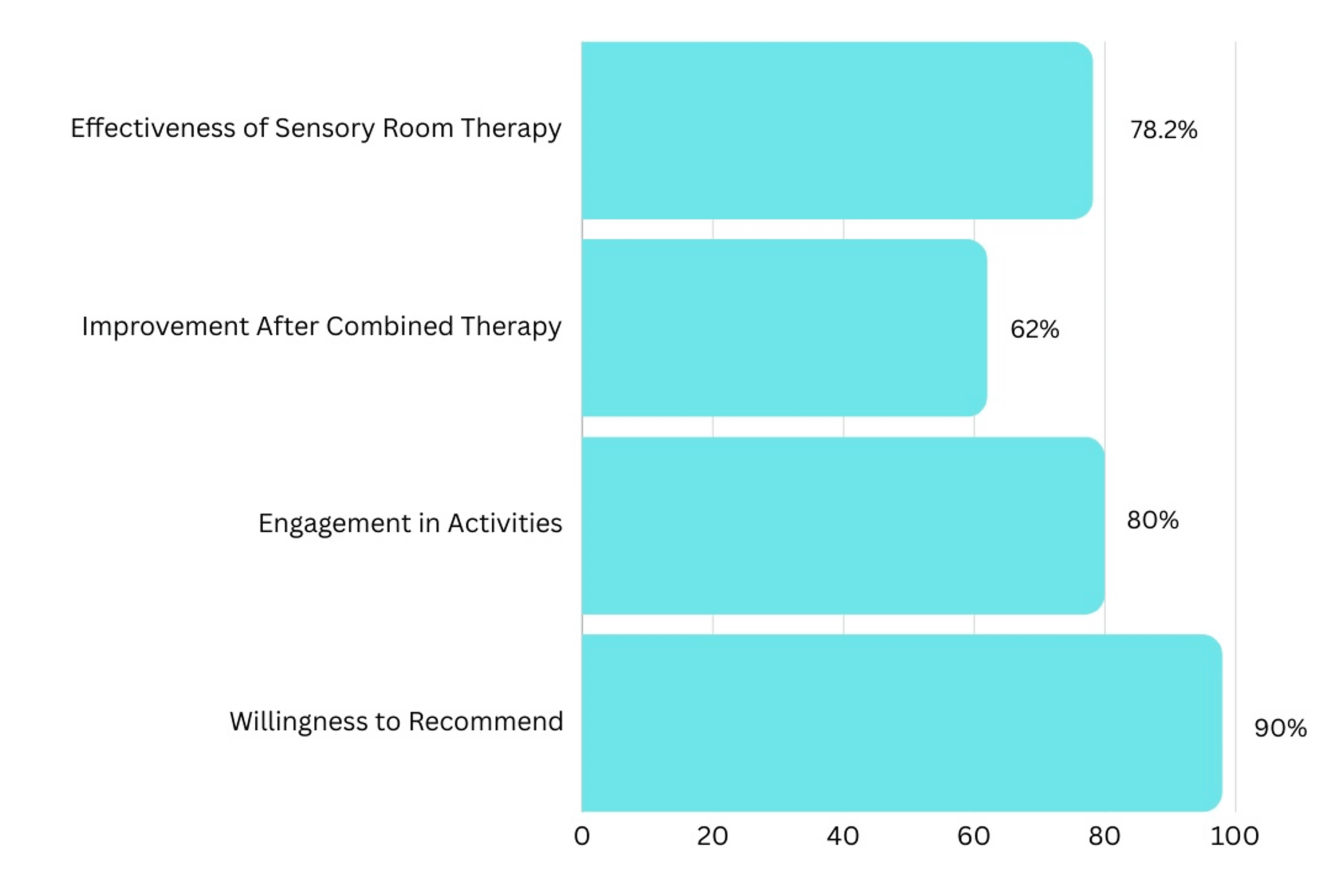
Efficiency of sensory room

A majority of parents (78.2%) rated sensory room therapy as highly effective. Furthermore, parents observed high to very high levels of improvement in their children following combined therapy (62%) compared to conventional therapy alone. Most children were moderately to highly engaged after this treatment and were actively participating in extracurricular activities (80%). Additionally, 98% of parents expressed willingness to recommend this therapy to other children with autism spectrum disorder (Figure 3).

## Discussion

The primary aim of this study is to compare the efficacy of sensory room therapy when combined with traditional therapy against traditional therapy alone. Following conventional therapy, the mean PCQIA values were relatively similar in both groups (p=0.222). However, the introduction of sensory room therapy led to a significant increase in the mean PCQIA score (p<0.001), underscoring the effectiveness of this supplementary treatment. Furthermore, sensory room therapy showed notable improvements in associated comorbidities and targeted symptoms, with minimal or no adverse effects. Consequently, the majority of parents endorsed it for other children with ASD.

Indeed, the demands of caring for children with ASD, coupled with limited availability, the high cost of specialized childcare, and parents' constrained capacity to maintain paid employment, have markedly diminished family productivity [12,13]. This was clearly evidenced in our study, where parents of children in group 2 exhibited lower levels of education and consequently lower family incomes compared to those in group 1. Additionally, the majority of children in both group 1 and group 2 exhibit poor spoken language, limited augmentative communication, and difficulties engaging in conversations with others. It is widely acknowledged that individuals with ASD commonly experience language challenges, with approximately 25% to 30% unable to speak verbally or only demonstrating moderate vocalization (using fewer than 30 words). Given the fundamental role of communication in daily life, any impediments in this area can lead to a range of adverse outcomes, including diminished academic performance, reduced quality of life, and behavioral issues [14]. Notably, communication abilities, language usage, levels of anxiety, and compulsive behaviors were found to be comparable between both groups, as were the therapies administered, except for ABA therapy, which exhibited a significantly higher utilization rate in group 1 (69.28%) compared to group 2 (36.36%), with a p-value of 0.012. Comparably, a study by Brignell et al. highlighted the effectiveness of intervention based on applied behavior analysis for autism spectrum disorder and indicated that ABA-based interventions primarily impact receptive language, adaptive behavior, and cognition [15].

In our study, we utilized the PCQIA score to assess the efficacy of sensory rooms in children receiving combined sensory therapy and conventional therapy compared to those receiving conventional therapy alone. Initially, PCQIA scores were similar between groups 1 and 2 following conventional therapy, with group 1 having a slightly higher mean PCQIA score of 34.1 compared to group 2's 33.40. However, upon the addition of sensory room therapy to the treatment regimen, the mean PCQIA score in group 2 increased significantly to 41.7, highlighting a notable variation in autism-associated challenges post-sensory room therapy. This finding aligns with the emphasis of the PCQIA score on social interaction and communication symptoms, particularly given that the social communication factor targets a larger number of items [11]. 

Furthermore, our study identified 236 patients experiencing medical conditions associated with autism disorder, with 125 of these suffering from digestive system issues. Previous research has indicated that common gastrointestinal complaints associated with ASD include chronic diarrhea, gaseousness, abdominal discomfort, and distension. Moreover, it has been observed that conditions such as reflux esophagitis and disaccharide malabsorption may contribute to the behavioral challenges experienced by non-verbal autistic patients [16].

To assess the efficacy of sensory rooms in treating ASD, caregivers were surveyed regarding their perception of sensory room therapy on their children. A significant 78.2% of caregivers reported high efficacy of sensory room therapy in addressing comorbidities observed during follow-up, with 62% expressing positive opinions towards this therapy. These parents noted a considerable improvement in targeted symptoms compared to conventional therapy. Research suggests that sensory-regulation-focused parent-child therapies can enhance parent-child interactions by reducing parental directedness and child sensory hypo-reactivity while increasing parental responsiveness [17,18]. Sensory integration-based interventions have shown promising outcomes in addressing sensory challenges and motor skills, while massage therapy has demonstrated improvements in sensory responses and ASD symptoms [19].

Following sensory therapy application, a notable 80% of children became either very engaged or engaged in extracurricular activities. Additionally, the vast majority of caregivers in group 1 (98%) endorsed recommending this therapy to other autistic patients. Recent evaluations highlight the benefits of sensory rooms, a form of sensory integration therapy (SIT), in improving sensory challenges, motor skills, and sensory responses in children with ASD [20]. These improvements are attributed to stress reduction and the enhancement of appropriate adaptive responses to sensory stimuli, concentration, and social interactions [21].

Occupational therapy protocols aim to guide children towards sensory-rich environments tailored to their individual sensory needs, with a focus on sensory integration. A 2021 meta-analysis by Randell et al. assessing the efficacy and safety of interventions targeting sensory challenges in children with ASD revealed that over 95% of occupational therapists incorporate sensory integration (SI) into their practice [22]. Kashefimerhr et al. observed that SI-based occupational therapy programs enhance non-functional behavior modification, communication, interaction skills, processing, motor abilities, and environmental adaptation in children [23].

Sensory interventions, according to sensory integration practitioners, offer numerous benefits, including improved focus in educational, therapeutic, and social settings, reduction of inappropriate behaviors such as self-harm, and enhancement of brain functioning in areas such as language and reading [24]. This approach is commonly employed to address developmental, behavioral, and learning challenges in children with ASD, ADHD, and developmental coordination disorders [25,26]. From a theoretical standpoint, the lack of sensory integration, as proposed by Ayer, may underlie behavioral issues observed in children with autism [24-26].

Overall, the findings of this study support the effectiveness of sensory room therapy as a complementary approach to conventional therapy in managing ASD in Lebanese children, emphasizing the importance of addressing sensory integration and medical comorbidities alongside behavioral interventions.

Impact of the study

This study emphasizes the significant advantages of integrating sensory room therapy with conventional therapy for children with ASD, underscoring its long-term effectiveness in improving sensory processing, communication skills, and overall behavioral outcomes. By demonstrating the value of a multi-sensory environment (MSE) in enhancing therapeutic interventions, this research fills a crucial gap in the literature, especially within the Lebanese context where data on such therapeutic approaches is scarce. The findings also serve to inform clinical practices and policies, advocating for the inclusion of sensory room therapy in treatment centers addressing the unique challenges faced by autistic children. Furthermore, the high parental satisfaction and endorsement of sensory room therapy highlight its practical benefits, potentially influencing future therapy standards and parental decision-making. These findings provide essential insights for healthcare authorities, encouraging the integration of sensory-based therapies into national autism care frameworks.

Study strengths

The study's strength lies in its longitudinal design, spanning over 10 years, which allowed for an in-depth examination of the combined therapy's efficacy in a real-world setting. The high parental satisfaction rates and the observed improvements in PCQIA scores after combining sensory room therapy with conventional methods reflect the practical success of this intervention. Additionally, this study represents one of the first comprehensive investigations into sensory room therapy in Lebanon, addressing a gap in existing literature and offering valuable data for future clinical practices.

Study limitations

Despite these strengths, certain limitations must be acknowledged. The study was conducted across eight centers in Lebanon, rather than the originally planned 15, due to external factors such as the COVID-19 pandemic, which led to the closure of several centers, and the ongoing economic crisis, which reduced patient compliance with various treatment regimens. Moreover, the lack of specialized centers dedicated solely to sensory or neurological treatments presented challenges, as we were unable to identify specific sensory or neurological abnormalities in our patient population. Access to comprehensive medical records was limited, and many parents were unaware of the specific results of their children's tests, which may have impacted the depth of our analysis. Additionally, interruptions in treatment due to the pandemic and economic hardship further hindered data collection.

Future directions

Future research should aim to overcome these limitations by expanding the number of participating centers and ensuring more comprehensive access to medical records for a better understanding of underlying neurological conditions. Additionally, further studies should explore strategies to improve data collection and control for variables such as phenotypic differences or variations in therapy dosages. Research into the scalability of sensory room therapy in other low-to-middle income countries could also provide further insights into its broader applicability. Lastly, addressing the impact of socioeconomic factors on therapy access and outcomes, as noted by the correlation between family income and treatment success, will be vital in shaping equitable healthcare strategies for ASD management in Lebanon and similar contexts.

## Conclusions

Sensory integration-based interventions demonstrate significant improvements in outcomes related to sensory challenges, motor skills, and ASD symptoms. Additionally, they contribute substantially to enhancing PCQIA. In Lebanon, it is evident that higher parental income positively impacts the efficacy of sensory room therapy.

Further studies on sensory integration therapy (SIT) are warranted, including trials comparing it to other therapies while controlling for time and attention. Moreover, investigations into phenotypic factors that influence treatment effects and testing different dosages are necessary. These findings can aid clinicians in personalizing interventions, selecting optimal dosages, and providing appropriate recommendations to families. Additionally, due to the unclear mechanism of action of SIT, more research is needed to understand how this intervention affects the underlying etiology of the disorder.
